# Abnormal blood lipid and electrocardiogram characteristics in common mental disorders

**DOI:** 10.1186/s12888-023-04965-9

**Published:** 2023-06-26

**Authors:** Yan Li, Chaohua Tang, Weibo Wu, Zhijian Li, Xuesong Li, Wei Huang, Wensheng Chen, Xiancong Mai, Xiaoling Li, Caixia Xu, Guojun Xie, Jiaquan Liang

**Affiliations:** Department of Psychiatry, The Third People’s Hospital of Foshan, Guangdong, People’s Republic of China

**Keywords:** Common mental disorders, Blood lipid, Electrocardiogram, QRS width, QTc

## Abstract

**Background:**

At present, there is not enough evidence to prove the relationship between blood lipid and electrocardiogram (ECG) abnormalities in common mental disorders (CMD). This study aimed to explore the relationship between them, to detect and prevent arrhythmia or sudden death.

**Methods:**

We collected 272 CMD patients (maintained a fixed drug dose pattern for 1 year or more), including 95 schizophrenias (SC), 90 bipolar disorders (BD) and 87 major depressive disorders (MDD), and 78 healthy controls (HC) from the Third People’s Hospital of Foshan, China. We analyzed and compared their blood lipid and ECG indicators, to clarify the relationship between them.

**Results:**

350 participants were included. There were no significant differences in age, gender, total cholesterol (TC), low density lipoprotein (LDL) and QTc (p > 0.05) among subjects. And there were significant differences in body mass index (BMI), triglyceride (TG), high density lipoprotein (HDL), heart rate, PR interval and QRS width (p < 0.05). Person correlation analysis showed that QRS width was positively correlated with BMI and TG. And negatively correlated with HDL. Meanwhile, QTc was positively correlated with BMI. Multiple linear regional analysis further proved that TG (B = 3.849, p = 0.007) and LDL (B = 11.764, p = 0.018) were the risk factors, and HDL (B = -9.935, p = 0.025) was the protective factor for QRS width increase.

**Conclusion:**

Long term medication of CMD patients should strengthen weight management, and conduct regular blood lipid and ECG examinations to achieve early detection and intervention in order to promote their health.

## Introduction

Common mental disorders (CMD) include major depressive disorders (MDD), bipolar disorders (BD), schizophrenias (SC), and so on [1, 2]. Long-term use of psychotropic drugs may affect heart function and cause abnormal lipid metabolism [3–5].

Studies have shown the correlation between mental illnesses such as MDD, BD, SC and obesity [6, 7]. On one hand, mental illnesses may lead to poor lifestyle choices such as an unhealthy diet and lack of exercise, resulting in obesity [8, 9]. On the other hand, obesity itself may cause mental health issues as weight gain can lead to self-image problems and further increase psychological stress. In addition, some anti-psychotic medications can also cause weight gain and abnormal blood lipid levels [10, 11], which may result in abnormal cardiovascular changes and increase the risk of sudden death [12–14].

ECG is cheap, convenient, fast, ready-made and non-invasive, therefore, it is important to regularly test the blood lipids and ECG [15–17]. Among the common ECG indicators (heart rate, PR, QRS width and QTc), QTc was considered one of the monitoring indicators for prevention of sudden death in patients with mental disorders [18, 19]. However, other ECG indicators were not paid much attention.

Thus, we included patients with CMD who had taken drugs steadily for one year or more. Through venous blood testing, we learned about their blood lipids situation, compared with various ECG indicators, and tried to clarify the relationship between them, which provided reference for patients’ health monitoring and clinical intervention.

## Methods

### Participants

This was a prospective study. Subjects with SC, BD and MDD were included in this study, who met the diagnostic criteria of the Diagnostic and Statistical Manual of Mental Disorders, Fourth Edition (DSM-IV) from July 2017 to September 2022 in the Third People’s Hospital of Foshan, Guangdong, China. Their age was ≥ 18 and < 45 years old. They were required to maintain a fixed drug dose pattern for 1 year or more before blood testing.

Healthy controls (HC): volunteers recruited through advertising in Foshan from March 2020 to December 2021; ①18–45 years old; ②No history or family history of psychosis was confirmed through an interview with a psychiatrist; ③Gender and age were matched with the patient group.

Exclusion criteria: ①Comorbidity of other mental disorders, such as intellectual disability, anxiety disorders, personality disorders, or other cognitive impairment, was assessed and excluded using the Structured Clinical Interview for DSM-IV (SCID); ②Patients with severe and unstable physical diseases, including severe liver and kidney function damage, cardiac insufficiency, diabetes, etc.; ③Smoking habits (≥ 1 cigarette per day) or drinking habits (≥ 1 unit alcohol per week); 1 unit alcohol = 480 ~ 600 ml of beer = 350 ml of low alcohol liquor or red wine, yellow wine = 50 ml of high spirits (that is 40 degrees or more) [2]; ④Did not cooperate with venous blood drawing, such as phobia, etc.

We obtained written informed consent from patients or their legal guardians. This study was approved by the ethics committee of the Third People’s Hospital of Foshan, China and the experiments were conducted following the declaration of Helsinki.

### Assessments

For the subjects who met the above conditions and were willing to participate in this study, after signed the informed consent, collected their names, gender and age through interviews. The height and weight of the subjects were evaluated by the nurses and the BMI (kg/m^2^) values of each subject were calculated.

Before drawing the venous blood of the subjects, they were required to be fasting for more than 8 h, and the nurses were required to complete the blood drawing from 7:30 to 10:00 in the morning. The night before blood drawing, they should maintain a normal diet, follow the previous work and rest, and should not drink alcohol or coffee after dinner. TC, TG, HDL and LDL were measured using the Mindray BS2000 Modular Analysis System (www.mindray.com/cn/products/laboratory-diagnostics/chemistry/modular-analysis-system/bs-2000 m) which Launched in 2013 and were recorded in the clinical data sheet of the subjects.

ECG measurement: ①Exposed the parts of the body that require electrodes, including both wrists and ankles. ②Limb leads were clamped to the corresponding limbs respectively, and the electrodes on the leads contacted the skin of the limbs. V1 was placed in the fourth intercostal space on the right edge of the sternum, V2 was located in the fourth intercostal space on the left edge of the sternum, V4 was located at the junction of the left clavicular midline and the fifth intercostal space, V3 was located between V2 and V4, V5 was located at the junction of the axillary front line and the fifth intercostal space, and V6 was located at the junction of the axillary midline and the fifth intercostal space. ③The ECG recorded the electrical activity of the heart for one minute, and the doctor in the ECG room read the chart and recorded the values of heart rate, PR interval, QRS width, and QTc.

### Data analyses

Statistical Product and Service Solutions 21 software (SPSS 21, https://www.ibm.com/analytics/spss-statisticssoftware) was used to analyze the data. Chi-square test and one way ANOVA were used to compare the differences in general demographic, then Bonferroni post-hoc analyses were performed. The relationships between BMI and variable indexes were analyzed by Pearson correlation, which adjusted for age and gender. Next, the obtained p values were then corrected by false discovery rate (FDR) correction. Taking heart rate, PR interval, QRS width, and QTc as dependent variables (Y), BMI, TG, TC, HDL and LDL as independent variables (X), age and gender as covariates, stepwise multiple linear regression models were established to analyze the effects of blood index components on ECG index.

## Results

### Comparison of demographic characteristics, metabolic indexes and ECG indexes

363 subjects took part in this study, including SC (n = 95), BD (n = 90), MDD (n = 87), and HC (n = 78), while 13 subjects were excluded due to consumption of breakfast before drawing blood. There were no significant differences in age, gender, TC, LDL and QTc (p > 0.05) among subjects. And there were significant differences in BMI, TG, HDL, heart rate, PR interval and QRS width (p < 0.05). Bonferroni post-hoc analyses showed that compared the BMI and blood lipid indexes of CMD and HC, we found that the BMI of MDD (p = 0.002) was significantly lower than that of HC. We also found that the BMI of SC (p < 0.001) and BD (p < 0.001) was significantly higher than that of MDD. What’s more, TG of SC (p = 0.021) was significantly higher than that of MDD. Our results indicated that HDL of SC was significantly lower than BD (p = 0.013) / MDD (p < 0.001) / HC (p = 0.016).

Compared the ECG indexes of CMD and HC, we found that the heart rate of CMD was increased, and PR interval of SC was significantly shorter than that of BD (p < 0.001) / MDD (p < 0.001) / HC (p < 0.001). Others, we found that QRS width increased significantly in SC. However, it is worth mentioning that the QTc of CMD patients was generally longer than HC, but there is no significant difference. (Table 1)

**Table 1:** Comparison of demographic characteristics, metabolic indexes and ECG indexes

	SC (n = 95)	BD (n = 90)	MDD (n = 87)	HC (n = 78)	F/χ^2^	p
Age (years)	32.46 ± 6.91	30.78 ± 8.22	29.74 ± 8.88	36.39 ± 12.44	2.55	0.055
Gender (male / female)	46/49	36/54	28/59	34/44	7.25	0.064
BMI(kg/m^2^) ^cde^	23.97 ± 4.22	23.68 ± 4.31	20.41 ± 3.48	22.65 ± 3.61	14.94	< 0.001*
TG(mmol/L) ^e^	1.70 ± 1.79	1.21 ± 0.75	1.05 ± 0.63	1.60 ± 2.32	3.71	0.012*
TC(mmol/L)	4.40 ± 0.81	4.37 ± 0.93	4.47 ± 0.98	4.70 ± 0.97	2.15	0.094
HDL(mmol/L) ^aef^	1.06 ± 0.31	1.23 ± 0.43	1.29 ± 0.38	1.23 ± 0.33	6.54	< 0.001*
LDL(mmol/L)	2.54 ± 0.66	2.547 ± 0.80	2.51 ± 0.77	2.57 ± 0.68	0.10	0.958
Heart rate (times/m) ^abcdf^	75.23 ± 12.85	82.85 ± 16.68	75.05 ± 13.58	67.98 ± 10.38	16.17	< 0.001*
PR interval (ms) ^acef^	113.51 ± 30.30	145.27 ± 20.34	142.24 ± 18.89	153.53 ± 19.49	51.17	< 0.001*
QRS width (ms) ^aef^	126.81 ± 31.27	91.34 ± 9.88	96.01 ± 9.58	97.48 ± 10.17	71.95	< 0.001*
QTc (ms)	417.55 ± 29.79	422.13 ± 21.75	416.94 ± 32.33	414.61 ± 22.19	1.10	0.350

BMI body mass index, TG triglyceride, TC total cholesterol, HDL high density lipoprotein, LDL low density lipoprotein; * indicated the comparison between groups (p < 0.05, two-tailed); Bonferroni post-hoc analyses: a: SC vs. HC, b: BD vs. HC, c: MDD vs. HC, d: MDD vs. BD, e: MDD vs. SC, f: BD vs. SC, p < 0.05, two-tailed; Value: mean ± standard deviation

### Pearson correlation between ECG and blood lipid

The results of Person correlation analysis showed that QRS width was positively correlated with BMI and TG. And negatively correlated with HDL. Meanwhile, QTc was positively correlated with BMI. (Table 2)

**Table 2:** Correlation between ECG and blood lipid

		Heart rate	PR interval	QRS width	QTc
BMI	r	0.090	0.045	**0.105**	**0.109**
	p	0.092	0.397	0.049*	0.041*
TG	r	-0.004	-0.104	**0.129**	-0.005
	p	0.946	0.053	0.016*	0.924
TC	r	0.007	0.015	-0.002	0.035
	p	0.893	0.784	0.967	0.520
HDL	r	0.036	0.067	**-0.157**	0.002
	p	0.507	0.213	0.003*	0.964
LDL	r	0.037	0.005	0.037	0.036
	p	0.486	0.931	0.492	0.502

BMI body mass index, TG triglyceride, TC total cholesterol, HDL high density lipoprotein, LDL low density lipoprotein; * indicated p < 0.05 (FDR correction), two-tailed

### Multiple linear regression analysis of heart rate, PR interval, QRS width and QTc

The stepwise multiple linear regression models (F_Heart rate_ = 1.733, p = 0.126; F _PR interval_ = 2.246, p = 0.051; F_QRS width_ = 3.626, p = 0.003; F_QTc_ = 0.986, p = 0.426) were established for analysis. Only the equation (taking QRs width as the dependent variable) passed the fitting requirement (p < 0.05). Finally, the elements entering the model were TG (B = 3.849, p = 0.007), HDL (B = -9.935, p = 0.025) and LDL (B = 11.764, p = 0.018). (Table 3)

**Table 3:** Multiple linear regression analysis of QRS width

Model	B	Standard error	Standard coefficient	t	p
(constant)	108.035	9.737	-	11.095	< 0.001*
BMI	0.199	0.319	0.036	0.624	0.533
TG	3.849	1.406	0.254	2.737	0.007*
TC	0.203	4.985	0.003	0.041	0.968
HDL	-9.935	4.411	-0.397	-2.252	0.025*
LDL	11.764	4.959	0.371	2.372	0.018*

BMI body mass index, TG triglyceride, TC total cholesterol, HDL high density lipoprotein, LDL low density lipoprotein; * indicated p < 0.05, two-tailed

## Discussion

Our study determined that the blood lipid and ECG indicators of CMD patients were significantly different from HC. Abnormal blood lipid was closely related to the abnormality of ECG. Moreover, HDL was the protective factor of QRs width increase.

Comparing the BMI and blood lipid indexes of CMD and HC, we found that the BMI of MDD was significantly lower than that of HC. It is known to us that antidepressants can cause nausea [5, 20]. At the same time, no appetite is the core symptom of MDD [21], resulting in weight loss, which leads to a decline in BMI. We also found that the BMI of SC and BD was significantly higher than that of MDD. This was consistent with our previous research results. Generally, CMD who received regular medication were more likely to be obese [2, 22]. What’s more, TG of SC was significantly higher than that of MDD. TG synthesized in vivo is mainly in the liver, followed by adipose tissue. Its main function is to supply and store energy, and it can also fix and protect internal organs. However, the increase of TG is related to diet structure and is the risk factor for cardiovascular disease [23, 24]. Previous studies have shown that the diet structure and intestinal flora of SC have specific changes [25–27], which made the patient’s diet tend to be unhealthy and result in leading to abnormal TG [28]. HDL is composed of proteins, lipids and their regulatory factors. HDL protein components include apolipoproteins, enzymes, lipid transfer proteins, acute phase response proteins, complement components and other protein components. It can transport the excess cholesterol in the blood to the liver, process and decompose it into cholic acid salts, and excrete it through the bile duct, thus forming a special way of blood lipid metabolism. It can enhance the ability of blood lipid metabolism and keep the blood vessels unblocked [29, 30]. Our results indicated that HDL of SC was significantly lower than BD/MDD/HC, which was related to the use of antipsychotic drugs [31, 32]. Meanwhile, SC itself also had HDL anomalies [33], and Hannah et al. further verified the metabolic abnormalities of SC at the genetic level, which also explained our experimental results [34].

Comparing the ECG indexes of CMD and HC, we found that the heart rate of CMD was increased, and PR interval of SC was significantly shorter than that of BD/MDD/HC. The heart is doubly innervated by sympathetic and parasympathetic nerves. The sympathetic nerve fiber terminals release norepinephrine (NE) and act on myocardial cells’ β1 adrenergic receptors (β1 receptors). Acetylcholine (Ach) released from parasympathetic nerve fiber terminals acts on cholinergic receptors (M receptors) in cardiomyocytes. Psychotropic drug receptors overlap with heart related receptors to some certain extent, thus affecting the change of heart rate and PR interval [35–37]. Others, we found that QRS width increased significantly in SC. In clinical practice, in order to better control the psychiatric symptoms of SC, patients need to take sufficient antipsychotic drugs during treatment, which seems to affect the cardiac function inevitably, thus increasing the risk of sudden death of SC [38]. However, it is worth mentioning that the QTc of CMD patients was generally longer than HC, but there is no significant difference. Previous studies showed that the QTc of SC was significantly prolonged [39], which was not completely consistent with our results. With the standardization of diagnosis and treatment for mental disorders, the first-line antipsychotic drugs for clinicians according to the International Clinical Guidelines for psychiatric related diseases are the second-generation antipsychotic drugs [40, 41], and SSRIS drugs were commonly used as antidepressants [42, 43], which have relatively little impact on patients’ heart QT interval. QTc prolongation can be reversible, depending on the underlying cause. The patients selected for this study were those who had been taking medication regularly in a stable phase. After long-term medication use, the heart can tolerate the effects of the medication, resulting in partial recovery of the QTc interval [41, 43, 44]. In addition, the subjects in this study had a long course of illness (≥ 1 year), regular medication treatment, and were mostly in stable stage of the disease. The risk of QTc prolongation in such patients is relatively low [45]. Therefore, these differences may be the reasons for the lack of significant differences in our results.

When we performed Pearson correlation analysis on blood lipid indicators and ECG indicators, the results showed that QRS width was positively correlated with BMI and TG, negatively correlated with HDL, and QTc was positively correlated with BMI. Multiple linear regional analysis determined that TG and LDL were the risk factors, and HDL was the protective factor for QRS width increase. QRS complex reflects changes in depolarization potential and time of left and right ventricles. The time from the starting point of QRS complex to its end point is the QRS time limit. Generally, the time limit of QRS complex is 0.06 ~ 0.10s [46]. The prolongation of QRS duration can be seen in ventricular hypertrophy, bundle branch block, preexcitation syndrome, and intraventricular differential conduction. Our results were consistent with previous studies. TG and LDL were recognized to damage the vascular environment [47], and HDL has a protective effect on cardiovascular system [48].

However, we know that there are many factors affecting blood lipid and ECG, including smoking, drinking, age, race, eating habits, etc. What’s more, our subjects were only from the South China, so we still need to be cautious when generalizing the conclusions.

Above all, long term medication of CMD patients is easy to lead to abnormal blood lipid and ECG. They should strengthen weight management, and conduct regular blood lipid and ECG examinations to achieve early detection and promote their health.

## Data Availability

The datasets generated and/or analyzed during the current study are not publicly available due to confidentiality but are available from the corresponding author on reasonable request.
